# Comparative Effectiveness and Safety of Intrauterine Contraception and Tubal Ligation

**DOI:** 10.1007/s11606-022-07433-4

**Published:** 2022-02-23

**Authors:** Eleanor Bimla Schwarz, Carrie A. Lewis, Melanie S. Dove, Eryn Murphy, Diana Zuckerman, Claudia Nunez-Eddy, Daniel J. Tancredi, Raegan McDonald-Mosley, Sarita Sonalkar, Mark Hathaway, Aileen M. Gariepy

**Affiliations:** 1 Division of General Internal Medicine, Department of Medicine at Zuckerberg San Francisco General Hospital and Trauma Center, UCSF, San Francisco, CA USA; 2 Center for Healthcare Policy and Research, UC Davis, Sacramento, CA USA; 3 National Center for Health Research, Washington, DC USA; 4 Planned Parenthood of Maryland, Baltimore, MD USA; 5 Perelman School of Medicine, University of Pennsylvania, Philadelphia, PA USA; 6 Reproductive Health and Family Planning, JHPIEGO, Washington, DC USA; 7 Yale Department of Obstetrics, Gynecology and Reproductive Sciences, New Haven, CT USA

**Keywords:** comparative effectiveness, intrauterine contraception, tubal ligation, permanent contraception, female sterilization, Medicaid, low income, disparities, reproductive justice

## Abstract

**Background:**

Tubal ligation remains common in the USA, especially among low-income patients.

**Objective:**

To compare the effectiveness and safety of intrauterine contraceptives (IUC) to laparoscopic tubal ligation for Medicaid clients.

**Design:**

We partnered with patient and clinician stakeholders to conduct a retrospective cohort study using California Medicaid claims for patients who had an IUC placed or laparoscopic tubal ligation performed in 2008–2014, excluding procedures performed within 42 days of a birth. We applied log-linear (Poisson) event-history regression models for clustered person-period data to adjust for sociodemographic variables and pre-procedure health status when examining associations between these contraceptive procedures and claims related to contraceptive failure, complications, and pain in the first year post-procedure.

**Key Results:**

We identified 35,705 patients who had a levonorgestrel IUC placed, 23,628 patients who had a copper IUC placed, and 23,965 patients who underwent laparoscopic tubal ligation. In unadjusted analyses, rates of pregnancy within 1 year were similar following levonorgestrel IUC (2.40%) or copper IUC placement (2.99%) or tubal ligation (2.64%). In adjusted analyses, compared to tubal ligation, pregnancy was less common following placement of a levonorgestrel IUC (adj IRR 0.72, 95% CI 0.64–0.82) and similar with placement of a copper IUC (adj IRR 0.92, 95% CI 0.82–1.05). Procedural complications such as infection (0.35% vs. 2.91%) were significantly less common with IUC placement than tubal ligation. Claims for pelvic and abdominal pain decreased in frequency with time since all procedures; 6 to 12 months post-procedure, pelvic pain claims were less common after levonorgestrel IUC (adj IRR 0.69, 95% CI 0.65–0.73) or copper IUC placement (adj IRR 0.70, 95% CI 0.66–0.75) than tubal ligation.

**Conclusions:**

IUC appears at least as effective as laparoscopic tubal ligation at 1-year post-procedure with lower rates of infection and pelvic pain 6 to 12 months post-procedure.

**Clinical Trial Registration:**

NCT03438682

**Supplementary Information:**

The online version contains supplementary material available at 10.1007/s11606-022-07433-4.

## INTRODUCTION

Effective family planning involves careful consideration of many aspects of available contraceptive methods.^1^ In a prior study, patients have highlighted the importance of clear communication about the relative effectiveness of available contraceptive methods.^2^ Unfortunately, misperceptions of contraceptive effectiveness are common.^3,4^

Historically, “typical use” failure rates have been calculated for short-acting contraceptive methods by analyzing data on rates of pregnancy within the first year of use, as reported to the National Survey of Family Growth (NSFG)^5^; these “typical use” failure rates are considerably greater than the “perfect use” failure rates reported in clinical trials of short-acting contraceptives. For long-acting contraceptives, “typical use” failure rates have been presumed to be equivalent to those reported in clinical trials. Thus, the US Food and Drug Administration states that after either tubal ligation or intrauterine contraceptive (IUC) placement, “less than 1 pregnancy is expected per 100 women.” However, in real-world clinical practice, the effectiveness and safety of many clinical procedures have been found to differ from what was seen in clinical trials.^6^ Further, the healthcare experiences of publicly and privately insured individuals in the USA differ in many meaningful ways.

Tubal ligation remains common in the USA, particularly among low-income individuals^7^ and those with chronic medical conditions such as diabetes.^8^ Compared to privately insured individuals, US patients with public insurance more frequently report a desire for reversal of tubal sterilization.^9^ As Medicaid offers only limited coverage of treatment for infertility, it is important that individuals considering permanent contraception are well informed about all of their contraceptive options. To inform such contraceptive counseling, we estimated the real-world effectiveness and safety of placement of a levonorgestrel or copper intrauterine contraceptive (IUC) as compared to laparoscopic tubal ligation among a large and diverse cohort of individuals receiving publicly funded healthcare in California.

## METHODS

With input from a stakeholder advisory board including patients and clinicians, we designed this retrospective cohort analysis of claims for Medicaid-funded tubal ligation and IUC placement procedures performed for Californian patients aged 18–50 between January 1, 2008, and August 31, 2014. Stakeholder engagement in this project was supported by regular meetings of a 10-person advisory board (which included 6 patients, 3 physicians, and a reproductive justice advocate) and a 12-person patient perspectives committee, which included a balance of women who had both positive and negative experiences with sterilization, a number of women who had been Medi-Cal clients at the time of their procedures, and some who had been involved with large online patient support groups. These patient stakeholders were involved in formulating research questions, designing this analysis, and interpreting the findings.

**Figure 1:**
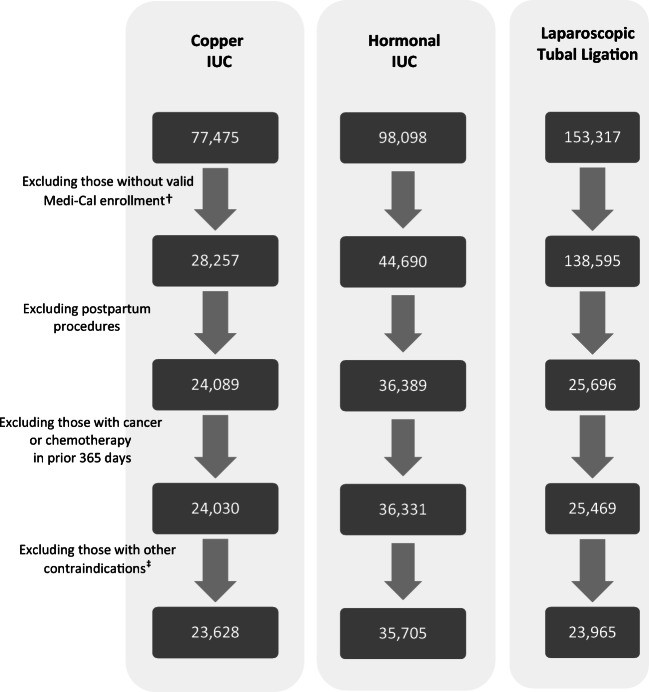
Long-acting contraceptive procedures performed for Medicaid clients in California, 2008–2014^*^.

We analyzed linked data obtained from the Centers for Medicare and Medicaid Services (CMS) Research Data Assistance Center (ResDAC), including claims, encounters, and eligibility information on recipients of California’s Medicaid program, Medi-Cal. Medi-Cal covers family planning services for enrollees who are eligible if their family income is at or below 138% of the federal poverty level (FPL); pregnancy-related Medi-Cal is available to those living in Californian at or below 213% FPL. As we were interested in comparing levonorgestrel and copper IUC, we excluded patients with claims for IUC of unknown type (*n* = 46,657). As postpartum women require different approaches for tubal ligation and IUC placement, we also excluded patients with postpartum contraceptive procedures (defined as those with any pregnancy claims in the 42 days prior to a procedure or 7 days post-procedure or any delivery claim within 180 days following procedure), and claims in the 2 years pre-procedure for any conditions that might have precluded general anesthesia which is needed for laparoscopic tubal ligation (i.e., congestive heart failure, chronic obstructive pulmonary disease, pulmonary hypertension, home oxygen) or placement of an IUC (i.e., cervical cancer, congenital uterine malformations). We also excluded those with claims indicating cancer in the year prior to tubal ligation, due to concerns that cancer treatments often decrease fertility (Fig. 1).

To estimate the real-world effectiveness of IUCs and tubal ligation for individuals receiving Medicaid-funded healthcare, we examined incidence rates of claims for pregnancy within 12 months post-procedure, censoring participants upon identification of any claims for hysterectomy, oophorectomy, pregnancy, infertility services or consultation, or the end of available data (12/31/14). We further censored participants at the time of a claim indicating IUC removal or any claims for a new or additional form of contraception. We limited this analysis to the first 12 months after IUC placement to reduce the possibility that subsequent gaps in Medicaid enrollment might result in misclassification of individuals whose IUC had been removed. When estimating rates of contraceptive failure, we excluded the < 1% of patients found to have a very early (i.e., a “luteal phase”) pregnancy not detected at the time of the contraceptive procedure (defined by pregnancy claim(s) within 30 days following contraceptive procedure or a delivery claim within 180 days following procedure). We used log-linear (Poisson) regression models for person-periods (nested within clusters) to examine associations between these long-acting contraceptive procedures and post-procedure pregnancy rates, adjusting for age group, race/ethnicity, region, year of procedure, months of Medi-Cal enrollment in the 2 years pre-procedure, and baseline health measures (evidenced by claims in the 2 years prior to the index contraceptive procedure indicating obesity, pregnancy (categorized as none, ectopic pregnancy, non-ectopic pregnancy), endometrial ablation, pelvic inflammatory disease, and any contraceptive claims). We selected this approach to modeling because it allows us to account parsimoniously for risk that varies over distinct and possibly censored follow-up periods.^10^ Because pregnancy is a qualifying condition for Medi-Cal, participants were not censored from analyses of contraceptive effectiveness if they had gaps in enrollment.

To examine the comparative safety of these methods, we tabulated the proportion of patients who had claims that might indicate a procedural complication within 30 days of procedure. For example, we used multiple ICD-9 codes (available from authors) to identify claims related to surgical site infections, fever, and septicemia which we categorized as “infection” and separately examined ICD-9 codes indicating “pelvic inflammatory disease.” After noting higher-than-expected rates of hysterectomy at the time of tubal ligation, we used a multivariable logistic model to examine variables associated with hysterectomy at the time of tubal ligation. We then tabulated the proportion of patients who had any claims indicating post-procedural pelvic pain, pelvic inflammatory disease, genitourinary pain, abdominal pain or gastrointestinal symptoms, non-abdominal pain, or abnormal uterine bleeding (the ICD-9 and CPT codes used to specify these variables are available from the authors by request). We calculated the number of days with one or more claims indicating each of these conditions per 100 patients days during each of the following time periods: (a) on the day of the procedure, (b) days 2–90, (c) days 91–180, and (d) days 181–365 post-procedure. For each of these time periods, we created separate generalized estimating equation (GEE Poisson) models to examine the significance of differences in the rate at which patients had claims for (a) pelvic pain, (b) pelvic inflammatory disease, (c) abnormal uterine bleeding, (d) abdominal pain or gastrointestinal symptoms, (e) genitourinary pain, and (f) non-abdominal pain in the year following their contraceptive procedure, adjusting all models for age group, race/ethnicity, region, year of procedure, Medi-Cal program funding procedure, Charlson comorbidity index,^6^ concomitant endometrial ablation, months of Medi-Cal enrollment in 2 years pre-procedure (log transformed), and claims in the 2 years pre-procedure indicating obesity, fibroids, pregnancy, pelvic pain, abdominal pain or gastrointestinal symptoms, genitourinary pain, non-abdominal pain, menorrhagia, pelvic inflammatory disease, and any contraceptive use, and censoring observation at the time of pregnancy, hysterectomy, oophorectomy, IUC removal, switch to another type of IUC, placement of a contraceptive implant or tubal ligation, enrollment gaps of more than 3 months, or end of data availability on December 31, 2014. This study was approved by the UC Davis Institutional Review Board as protocol 1074677-5.

## RESULTS

We identified 35,705 patients who had a levonorgestrel IUC placed, 23,628 patients who had a copper IUC placed, and 23,965 patients who underwent laparoscopic tubal ligation. The demographic characteristics of Californian patients with Medicaid-funded claims for laparoscopic tubal ligation or an IUC placement more than 42 days following any births are shown in Table 1. On average, patients with claims for tubal ligation were 32.8 years of age while those with claims for IUC placement were younger (26.5 years for levonorgestrel and 27.2 years for copper IUC). The healthcare utilization of patients receiving tubal ligation or IUC varied in the 2 years prior to their contraceptive procedure; those who had tubal ligation were less likely to have pregnancy-related claims in the prior 2 years and more likely to have claims related to obesity (Table 2). Endometrial ablation was performed on the same day as laparoscopic tubal ligation for 2.41% of patients compared to < 0.001% of those who had an IUC placed.

**Table 1:** Sociodemographic Characteristics of California Patients Receiving Medicaid-Funded Long-Acting Contraceptive Procedures, 2008–2014

	**Levonorgestrel IUC (*n*= 35,705)**	**Copper IUC (*n*= 23,628)**	**Tubal ligation (*n*= 23,965)**
**Age**, years	26.53 **±** 6.51	27.24 **±** 6.61	32.8 **±** 6.7
**Age category**			
18–27 years	64.55	58.70	23.54
28–33 years	21.18	23.43	33.61
34–44 years	12.59	16.56	37.33
45–50 years	1.68	1.32	5.52
**Race-ethnicity**			
White, non-Hispanic	27.50	21.42	31.32
Black, non-Hispanic	10.65	7.41	8.25
Hispanic	48.29	54.17	50.04
Asian	4.63	7.10	3.33
Other^*^	8.93	9.90	7.05
**Region of residence**			
Northern/Sierra	4.30	2.74	8.11
Sacramento Area	11.92	7.00	8.96
San Joaquin Valley	27.57	24.86	23.28
Greater Bay Area	18.89	16.48	7.53
Central Coast	4.67	4.15	5.50
Los Angeles	11.98	23.06	18.04
Southern California outside Los Angeles	20.68	21.71	28.57
**Medi-Cal Program covering procedure**			
Fee for service	5.92	5.95	10.93
Managed care	94.08	94.05	89.07
**Year of index procedure**			
2008	16.94	18.52	16.30
2009	16.85	16.43	15.30
2010	12.84	16.62	15.23
2011	14.45	14.39	14.70
2012	16.19	12.47	13.23
2013	13.19	12.31	14.00
2014^†^	9.55	9.25	11.24
**Endometrial ablation**^‡^	*n* < 11	*n* < 11	2.41
**Months of Medi-Cal enrollment after procedure**	32.84 **±** 21.41	34.31 **±** 21.89	33.57 **±** 21.91

Excluding procedures performed within 42 days of a birth. Table presents column percentages or mean **±** standard deviation. *P* < 0.001 for all comparisons across columns

^*^Native American/Hawaiian, Pacific Islander, multi-race, unknown

^†^Through 8/31/2014

^‡^Within 7 days of contraceptive procedure; CMS policy advises limiting detail on cells with *n* < 11

**Table 2:** Pre-procedural Health Care Utilization of California Patients Receiving Medicaid-Funded Long-Acting Contraceptive Procedures, 2008–2014

	**Levonorgestrel IUC (*n* = 35,705)**	**Copper IUC (*n* = 23,628)**	**Tubal ligation (*n* = 23,965)**
Obesity (BMI ≥ 30 kg/m^2^)	9.14	8.77	11.70
Any pregnancy	70.94	74.95	57.65
C-section	18.40	20.56	10.85
Ectopic pregnancy	1.31	1.60	1.71
Any contraceptive use	51.51	52.51	49.10
Fibroids	1.72	0.94	6.53
Mood	11.32	9.84	16.21
Pelvic pain	15.32	14.66	35.25
Pelvic inflammatory disease (PID)	1.49	1.45	4.56
Menorrhagia	28.66	28.67	33.82
Abdominal pain and gastrointestinal symptoms	31.69	30.82	42.51
Genitourinary pain	4.46	4.08	6.25
Non-abdominal pain	30.71	29.65	41.11
Charlson Comorbidity Index	0.22 **±** 0.62	0.22 **±** 0.65	0.37 **±** 0.80
Months of Medi-Cal enrollment in 2 years prior to procedure	18.00 **±** 6.86	18.45 **±** 6.64	19.20 **±** 6.70

Excluding procedures performed within 42 days of a birth, through 8/31/2014. The percentage of patients with one or more claims related to various conditions in 2 years before their contraceptive procedure. *P* < 0.001 for all variables compared across the three contraceptive procedures. Data are mean **±** standard deviation or percentages.

*BMI* body mass index

In unadjusted analyses, rates of pregnancy within 1 year were similar for patients who had a levonorgestrel IUC placement (2.40%), copper IUC placement (2.99%), or laparoscopic tubal ligation (2.64%) per 100 woman-years of observation. In adjusted analyses (Table 3), compared to tubal ligation, pregnancy within 1 year was less common following placement of a levonorgestrel IUC (adj IRR 0.72, 95% CI 0.64–0.82) and similar with placement of a copper IUC (adj IRR 0.92, 95% CI 0.82–1.05). In adjusted analyses, rates of pregnancy within 1 year were lower following levonorgestrel compared to copper IUC placement (adj IRR 0.78, 95% CI 0.70–0.87). Claims related to ectopic pregnancy were less common following laparoscopic tubal ligation (0.29%) or placement of a hormonal (0.63%) or copper (0.83%) IUC than in the years prior to these procedures (Table 2).

**Table 3:** Pregnancy-Related Claims Within 12 Months of Intrauterine Contraceptive Placement Compared to Tubal Ligation

	Unadjusted incidence rate per 100 woman-years (95% CI)	Unadjusted incident rate ratio (95% CI)	Adjusted^*^ incident rate ratio (95% CI)
Laparoscopic tubal ligation	2.64 (2.43–2.86)	Referent	Referent
Levonorgestrel IUC	2.40 (2.23–2.59)	0.91 (0.82–1.02)	**0.72 (0.64–0.82)**
Copper IUC	2.99 (2.76–3.24)	**1.13 (1.01–1.27)**	0.92 (0.82–1.05)

Pregnancy-related claims exclude luteal-phase pregnancies, defined as any pregnancy claims within 30 days following tubal ligation or IUC procedure or any delivery claim within 180 days following the procedure; this study focused on contraceptive procedures performed more than 42 days following any births.

^*^Generalized estimating equations for multivariable Poisson regression, adjusted for age group, race/ethnicity, region, year of procedure, endometrial ablation within 7 days of tubal ligation, Medi-Cal program, months of Medi-Cal enrollment in 2 years pre-procedure (log transformed), and baseline health measures (evidenced by claims in 2 years prior to the index contraceptive procedure indicating obesity, pregnancy (categorized as none, ectopic pregnancy, non-ectopic pregnancy), pelvic inflammatory disease, Charlson comorbidity index, and any contraceptive claims. We censored participants from these analyses at the time of any claims for IUC removal, initiation of another form of contraception, pregnancy, or luteal phase, infertility services or consultation, hysterectomy, oophorectomy, or end of data availability on 12/31/2014

Values shown in bold are statistically significant, at the level of *p* < 0.05

Complications and additional procedures were significantly more common within 30 days of laparoscopic tubal ligation than IUC placement (Table 4). Acute hemorrhage complicated less than 0.03% of IUC placements but 0.80% of tubal ligations. Similarly, claims related to infections were less common following IUC placement than tubal ligation (0.35% vs 2.91%). Hysterectomy, which may have been planned in advance, was performed at the time of 3.96% of tubal ligations but less than 0.01% of IUC placements. After adjusting for covariates, we found that hysterectomy performed at the time of tubal ligation most commonly involved younger patients, and those who had claims for fibroids and/or menorrhagia in the 2 years prior to their procedure, and/or who had an endometrial ablation on the day of their procedure (data not shown in tables).

**Table 4:** Medical and Surgical Complications and Additional Procedures Performed Within 30 Days of Placement of a Medicaid-Funded Long-Acting Contraceptive Procedure Provided to California Patients, 2008–2014

	Levonorgestrel IUC (*n* = 35,705)	Copper IUC (*n* = 23,628)	Laparoscopic tubal ligation (*n* = 23,965)
Medical complications^*^			
Acute cardiovascular event	15 (0.04%)	< 11 (< 0.01%)	41 (0.17%)
Deep vein thrombosis or pulmonary embolism	16 (0.04%)	13 (0.06%)	28 (0.12%)
Anesthetic/respiratory complications	57 (0.16%)	30 (0.13%)	172 (0.72%)
Infection	112 (0.31%)	93 (0.39%)	698 (2.91%)
**Surgical complications**			
Acute hemorrhage	12 (0.03%)	< 11 (< 0.01%)	192 (0.80%)
Abdominal injury	124 (0.35%)	65 (0.28%)	713 (2.98%)
Cervical laceration	< 11 (< 0.01%)	0 (0%)	15 (0.06%)
**Additional procedures**			
Hysterectomy^†^	< 11 (< 0.01%)	0 (0%)	948 (3.96%)
Salpingectomy^†^	0 (0%)	0 (0%)	950 (3.96%)
Oophorectomy^†^	< 11 (< 0.01%)	0 (0%)	1887 (7.87%)
Laparoscopy	15 (0.04%)	< 11 (< 0.01%)	335 (1.40%)
Hysteroscopy	36 (0.10%)	< 11 (< 0.01%)	922 (3.85%)

Excluding procedures performed within 42 days of a birth. Statistically significant with *p* < 0.05 for all comparisons of tubal ligation to IUC shown in the table. Data are *n* (%); CMS guidance limits presentation of cells with *n* less than 11

^*^ICD-9 codes used to define each variable are available from authors by request

^†^Claims data do not allow identification of whether additional procedures were planned in advance

Claims for pelvic, abdominal, and genitourinary pain decreased with time following both IUC placement and tubal ligation. However, more than 6 months post-procedure, claims for such pain were significantly more common following tubal ligation than IUC placement, after adjusting for claims related to pain in the 2 years prior to the index procedure and censoring observation at the time of hysterectomy or oophorectomy (Table 5). No significant difference was appreciated in rates of pelvic inflammatory disease more than 6 months post-procedure.

**Table 5:** Claims Related to Pain and Menstrual Bleeding in the Year Following Long-Acting Contraceptive Procedures Provided by Medicaid to California Patients, 2008–2014

	**Day of procedure**		**2–89 days**			**90–179 days**			**180–364 days**		
	%^*^rate^†^	**Adj IRR**^‡^ **(95% CI)**	%^*^	**Rate**^†^	**Adj IRR**^‡^ **(95% CI)**	%^*^	**Rate**^†^	**Adj IRR**^‡^ **(95% CI)**	%^*^	**Rate**^†^	**Adj IRR**^‡^ **(95% CI)**
**Pelvic pain**											
Tubal ligation	19.41	Ref	11.52	0.21	Ref	6.63	0.13	ref	9.61	0.12	Ref
Levonorgestrel IUC	0.55	**0.06** **(0.05–0.07)**	4.52	0.07	**0.55** **(0.52–0.59)**	3.41	0.06	**0.64** **(0.59–0.68)**	6.16	0.05	**0.69** **(0.65–0.73)**
Copper IUC	0.32	**0.04** **(0.03–0.05)**	3.92	0.06	**0.51** **(0.48–0.55)**	3.13	0.04	**0.56** **(0.51–0.61)**	5.68	0.05	**0.70** **(0.66–0.75)**
**Menstrual bleeding concerns**											
Tubal ligation	6.71	Ref	4.87	0.08	Ref	3.91	0.07	Ref	5.67	0.06	Ref
Levonorgestrel IUC	3.64	**1.35** **(1.23–1.48)**	5.22	0.07	**1.45** **(1.35–1.56)**	3.04	0.04	**0.84** **(0.77–0.92)**	4.98	0.03	**0.72** **(0.67–0.77)**
Copper IUC	2.64	1.09 (0.98**–**1.22)	4.21	0.06	**1.18** **(1.09–1.29)**	3.37	0.04	0.93 (0.84**–**1.02)	6.10	0.04	0.96 (0.88**–**1.03)
**Abdominal pain and gastrointestinal symptoms**											
Tubal ligation	3.93	Ref	12.51	0.27	Ref	8.59	0.18	Ref	13.62	0.18	Ref
Levonorgestrel IUC	0.14	**0.05** **(0.04–0.07)**	5.78	0.10	**0.57** **(0.54–0.60)**	5.53	0.10	**0.80** **(0.76–0.85)**	9.84	0.10	**0.77** **(0.73–0.80)**
Copper IUC	0.19	**0.08** **(0.06–0.11)**	5.95	0.10	**0.61** **(0.58–0.65)**	4.95	0.09	**0.76** **(0.71–0.81)**	9.19	0.09	**0.80** **(0.76–0.84)**
**Non-abdominal pain**											
Tubal ligation	0.35	Ref	12.69	0.29	Ref	14.04	0.34	Ref	20.81	0.34	Ref
Levonorgestrel IUC	0.26	1.31 (0.94**–**1.82)	9.11	0.19	**1.07** **(1.02–1.11)**	9.06	0.19	**0.89** **(0.86–0.93)**	14.69	0.19	**0.90** **(0.87–0.92)**
Copper IUC	0.28	**1.58** **(1.11–2.26)**	8.29	0.16	1.03 (0.99**–**1.08)	8.18	0.17	**0.91** **(0.86–0.95)**	13.28	0.16	**0.83** **(0.80–0.86)**
**Pelvic inflammatory disease**											
Tubal ligation	6.28	Ref	1.70	0.04	Ref	0.50	0.007	Ref	0.74	0.006	Ref
Levonorgestrel IUC	00.02	**0.01** **(0.00–0.01)**	0.47	0.01	**0.34** **(0.28–0.41)**	0.32	0.005	0.99 (0.75**–**1.30)	0.55	0.004	0.83 (0.66**–**1.04)
Copper IUC	0.01	**0.004** **(0.00–0.01)**	0.41	0.01	**0.35** **(0.28–0.43)**	0.34	0.005	1.12 (0.83**–**1.52)	0.55	0.004	0.95 (0.74**–**1.21)
**Genitourinary pain**											
Tubal ligation	0.13	Ref	1.36	0.02	Ref	1.21	0.02	Ref	2.05	0.023	Ref
Levonorgestrel IUC	0.06	0.62 (0.32**–**1.21)	0.82	0.01	0.92 (0.79**–**1.07)	1.76	0.01	0.86 (0.73**–**1.01)	1.41	0.012	**0.80** **(0.70–0.90)**
Copper IUC	0.03	0.47 (0.20**–**1.11)	0.72	0.01	0.84 (0.71**–**1.00)	1.69	0.01	0.85 (0.71**–**1.02)	1.16	0.011	**0.75** **(0.65–0.87)**

*Ref* referent

^*^Proportion of patients with one or more claims for condition during interval of interest; data censored on 8/31/2014

^†^Number of days with one or more claims for condition during interval per 100 patients days, rates were censored at time of claims indicating IUC removal or switch to another type of IUC, contraceptive implant or tubal ligation, pregnancy, hysterectomy, oopherectomy, enrollment gaps > 3 months, or end of data availability on December 31, 2014

^‡^Incident rate ratios from multivariable Poisson models adjusted for year of procedure, Medi-Cal program, age category, race/ethnicity, region, baseline months eligibility, endometrial ablation, health care utilization in the 2 years pre-procedure (i.e., claims related to abdominal pain or other gastrointestinal symptoms, genitourinary pain, menorrhagia, non-abdominal pain, pelvic inflammatory disease, pelvic pain, fibroids, mood disorder, obesity, pregnancy history, use of any contraceptive method, Charlson Comorbidity Index), censored at time of claims indicating IUC removal, switch to another type of IUD, placement of contraceptive implant or tubal ligation, pregnancy, hysterectomy, oophorectomy, gaps in enrollment >3 months, or end of available data on December 31, 2014

Values shown in bold are statistically significant, at the level of *p* < 0.05

Patients’ age and race/ethnicity impacted the frequency of claims for pain after tubal ligation or IUC placement. More than 6 months after their contraceptive procedure, younger patients were more likely to have claims for pelvic and abdominal pain (Table 6). Asian, Black, and Hispanic patients were significantly less likely to have claims related to pain 6 months after their contraceptive procedures, although they were as likely to have claims for menorrhagia or pelvic inflammatory disease as White patients (Table 6). As expected, pre-procedural clinical variables were also associated with post-procedural claims related to pain, pelvic inflammatory disease, and menstrual bleeding (Appendix).

**Table 6:** Associations Between Age, Ethnicity, and Number of Days with One or More Claims for Outcomes of Interest, 180–364 Days After Tubal Ligation or IUC Placement Provided by Medicaid to California Patients, 2008–2014

	Adjusted^*^incident rate ratios for outcomes of interest					
	**Pelvic pain**	**GI pain**	**Non-abdominal pain**	**GU pain**	**Abnormal uterine bleeding**	**PID**
**Age** (referent = 45+ years)						
18–27 years old	**1.71** **(1.51–1.94)**	**1.11** **(1.01–1.21)**	**0.54** **(0.51–0.57)**	0.85 (0.67**–**1.08)	0.88 (0.76**–**1.02)	**5.80** **(2.64–12.72)**
28–33 years old	**1.43** **(1.27–1.62)**	1.01 (0.93**–**1.11)	**0.67** **(0.63–0.71)**	0.92 (0.73**–**1.15)	**0.80** **(0.70–0.93)**	**3.95** **(1.80–8.65)**
34–44 years old	**1.31** **(1.16–1.47)**	0.97 (0.90**–**1.06)	**0.76** **(0.72–0.80)**	0.81 (0.64**–**1.01)	**0.86** **(0.75–0.98)**	**2.85** **(1.31–6.21)**
**Race/ethnicity** (referent = White)						
Asian	**0.64** **(0.56–0.74)**	0.79 (0.72**–**0.88)	**0.54** **(0.52–0.61)**	0.68 (0.52**–**0.89)	**1.10** **(0.96–1.26)**	**0.76** **(0.43–1.32)**
Black	**0.91** **(0.84–0.98)**	0.87 (0.82**–**0.93)	**0.80** **(0.77–0.84)**	0.50 (0.40**–**0.61)	**0.91** **(0.82–1.02)**	**1.33** **(0.99–1.78)**
Hispanic	**0.76** **(0.72–0.80)**	0.93 (0.89**–**0.97)	**0.74** **(0.72–0.76)**	0.72 (0.64**–**0.80)	**0.97** **(0.90–1.04)**	**0.84** **(0.68–1.05)**
Other^†^	**0.83** **(0.76–0.91)**	1.01 (0.94**–**1.08)	**0.86** **(0.82–0.90)**	0.70 (0.57**–**0.85)	**1.05** **(0.94–1.17)**	**1.15** **(0.83–1.60)**

Excluding procedures performed within 42 days of a birth

*GI* gastrointestinal, *GU* genitourinary, *PID* pelvic inflammatory disease

^*^Incident rate ratios from multivariable Poisson models adjusted for age category, year of procedure, race/ethnicity, region, MediCal program, baseline months eligibility (log transformed), same-day ablation, health care utilization in the 2 years pre-procedure (i.e., claims related to abdominal pain or other gastrointestinal symptoms, genitourinary pain, menorrhagia, non-abdominal pain, pelvic inflammatory disease, pelvic pain, fibroids, mood disorder, obesity, pregnancy history, use of any contraceptive method, Charlson Comorbidity Index)

^†^American Indian, Alaska Native, Native Hawaiian, Asian Pacific Islander, multiracial, or race unknown

Values shown in bold are statistically significant, at the level of *p* < 0.05

## DISCUSSION

In this analysis of claims data from over 83,000 Californian patients who received publicly funded contraceptive services, we found that the typical-use contraceptive failure rate, as indicated by claims for pregnancy within 1 year of placement of a levonorgestrel IUC, was lower than that following laparoscopic tubal ligation, while pregnancy rates following copper IUC placement were similar to those following laparoscopic tubal ligation. These findings are important because IUC placement was far less likely to result in procedural complications than laparoscopic tubal ligation. Of note, this was the case even after we excluded from this analysis patients who are at highest risk of procedural complications, such as those with claims indicating congestive heart failure, chronic obstructive pulmonary disease, pulmonary hypertension, or need for home oxygen. Further, patients who underwent IUC placement were significantly less likely to have claims for pelvic, abdominal, or genitourinary pain 6 to 12 months post-procedure, even after adjusting for pain-related claims in the 2 years prior to the index procedure and censoring observation at the time of hysterectomy or oophorectomy. This may be because IUC users avoid the scar tissue and risk of adhesions that follow tubal ligation, and because hormonal IUC reduce menstrual cramping and flow.

Typical-use rates of pregnancy within the year following both tubal ligation and IUC placement were higher in California’s Medicaid program than many would expect, at over 2%. In a clinical trial that followed 58,324 European patients with a newly placed IUC for 1 year, only 118 (0.2%) contraceptive failures were identified (26 levonorgestrel, 92 copper), producing a Pearl index of 0.06 (95% CI: 0.04–0.09) for the levonorgestrel IUC and 0.52 (95% CI: 0.42–0.64) for copper IUCs.^11^ Although we found higher rates of pregnancy overall, perhaps due to differences in the sexual activity or fecundability of this population compared to the clinical trial participants, we similarly found that pregnancy rates were lower in the one year following placement of a levonorgestrel IUC than a copper IUC. In the U.S. Collaborative Review of Sterilization (CREST) study on surgical sterilization, the cumulative 10-year probability of pregnancy was less than 0.75%^12^; however, current clinical practice for California Medicaid patients appears to differ from that of the centers that participated in this landmark study more than 40 years ago. Whether this is due to variation in tubal ligation techniques or the populations served, as rates of obesity in the USA have increased dramatically over the last 40 years, deserves further study.

As desire for reversal of tubal ligation is known to occur,^13^ it is important that all patients considering tubal ligation receive thorough counseling regarding the comparative safety and effectiveness of IUC prior to undergoing tubal ligation. As the effectiveness and safety of many clinical procedures vary by patient comorbidity and clinician experience, the millions of US individuals who receive Medicaid-funded healthcare should be specifically informed about the comparative safety and effectiveness of IUC compared to tubal ligation provided to Medicaid clients, because desire for tubal reversal has been reported to be more common among Medicaid clients,^9^ and Medicaid coverage of infertility treatment is very limited in most states.

Strengths of this study include the large and diverse population studied with input from both patients and clinician stakeholders. When estimating rates of contraceptive failure, the fact that California covers abortion services for those eligible for Medicaid is an additional strength. Given federal restrictions on abortion funding, ResDAC includes very little abortion data. However, we believe that we were able to identify many pregnancies that were aborted using claims related to pregnancy testing and imaging which are included in ResDAC without subsequent claims for delivery, ectopic pregnancy, or management of early pregnancy loss. Further, undercounting of abortions should not differ with respect to type of contraceptive procedure performed prior to an undesired pregnancy. Additional limitations of this study include the fact that claims data do not allow identification of whether patients had an IUC in their uterus at the time they became pregnant; it is therefore possible that some patients expelled or removed their IUC prior to becoming pregnant. If our data includes a considerable number of patients who self-removed or expelled their IUC and did not start another form of contraception prior to becoming pregnant, then the typical-use failure rate we report here for the levonorgestrel IUC is higher than it should be, and patients may be even less likely to become pregnant in the first year following placement of a levonorgestrel IUC than following laparoscopic tubal ligation. In addition, this claims data does not provide detail on what type of laparoscopic tubal ligation was performed. We recognize that some salpingectomies and oophorectomies performed at the time of tubal ligation may have been planned with the goal of reducing risk of malignancy, while hysterectomies may have been planned due to a history of fibroids and menorrhagia. Although all analyses were adjusted for age and claims related to obesity and pain in the 2 years pre-procedure, our finding of more pelvic pain following tubal ligation than IUC may reflect continuation of conditions that made laparoscopy and tubal ligation a more likely contraceptive choice. Healthcare claims are an imperfect measure of health status as people who chose not to or could not access Medi-Cal-funded healthcare during the study period may have skewed baseline health measures. As such, claims data do not allow for ideal adjustment for baseline differences between groups. Further, observational studies can only identify associations, not causal relationships. Finally, due to the limited number of individuals with continuous enrollment for more than 1 year, we were unable to examine the comparative safety and effectiveness of these methods with extended use. In addition, it is possible that some patients may have had private insurance or received healthcare in another state during a gap in Medi-Cal enrollment.

Given growing interest in the option of early postpartum IUC placement, information about the comparative safety and effectiveness of postpartum tubal ligation and postpartum IUC placement remains needed, particularly as the majority of Medicaid-funded tubal ligation procedures performed in California are postpartum procedures. In addition, future studies are needed to clarify the comparative effectiveness and safety of long-acting contraceptives after the first year of use. In conclusion, placement of IUC appears at least as effective as laparoscopic tubal ligation for Medicaid clients at 1 year post-procedure with lower rates of procedural complications and pelvic pain 6 to 12 months post-procedure.

## Supplementary Information

ESM 1 (DOCX 22 kb)

## Declarations

### Disclaimer

The statements presented in this article are solely the responsibility of the authors and do not necessarily represent the views of the PCORI®, its Board of Governors, or its Methodology Committee.
