# Supplementary material for: Comparative Effectiveness and Safety of Intrauterine Contraception and Tubal Ligation
Source: J Gen Intern Med. 2022 Feb 23;37(16):4168–75. doi: 10.1007/s11606-022-07433-4 (PMC8863411; doi:10.1007/s11606-022-07433-4)
Supplement: Supplementary file 1 — (DOCX 22 kb) [file 11606_2022_7433_MOESM1_ESM.docx]

**Appendix: Pre-procedural clinical variables associated with number of days with one or more claims for each outcome of interest, 180-364 days after laparoscopic tubal ligation or IUC placement provided by Medicaid to California patients, 2008-2014**

|  | **Adjusted* Incident Rate Ratios for Outcomes of Interest** | | | | | |
| --- | --- | --- | --- | --- | --- | --- |
| **Pre-procedural** | **Pelvic Pain** | **Abdominal pain** | **Abnormal uterine bleeding** | **Nonabdominal pain** | **Pelvic Inflammatory Disease** | **Genitourinary pain** |
| Pelvic pain | **2.34**  **(2.22-2.46)** | **1.56**  **(1.50-1.62)** | **1.37**  **(1.28-1.47)** | **1.40**  **(1.36-1.44)** | **1.68**  **(1.36-2.07)** | **1.71**  **(1.53-1.91)** |
| Abdominal pain | **1.75**  **(1.67-1.84)** | **2.34**  **(2.25-2.43)** | **1.39**  **(1.31-1.48)** | **1.35**  **(1.31-1.38)** | **1.56**  **(1.28-1.91)** | **1.63**  **(1.45-1.82)** |
| Genitourinary pain | **1.20**  **(1.12-1.30)** | **1.61**  **(1.53-1.70)** | 1.09  (0.98-1.22) | **1.24**  **(1.19-1.29)** | **1.71**  **(1.31-2.24)** | **7.10**  **(6.37-7.90)** |
| Abnormal bleeding | **1.12**  **(1.07-1.18)** | 1.02  (0.98-1.06) | **1.96**  **(1.85-2.08)** | **1.05**  **(1.02-1.08)** | 1.06  (0.88-1.28) | **1.13**  **(1.02-1.26)** |
| Non-abdominal pain | **1.53**  **(1.46-1.61)** | **1.51**  **(1.45-1.57)** | **1.26**  **(1.18-1.34)** | **2.84**  **(2.76-2.93)** | **1.46**  **(1.20-1.77)** | **1.30**  **(1.17-1.45)** |
| Fibroids | **1.28**  **(1.16-1.40)** | **1.09**  **(1.01-1.18)** | **1.26**  **(1.12-1.43)** | 1.03  (0.98-1.09) | **1.98**  **(1.37-2.87)** | 1.09  (0.88-1.35) |
| PID | **1.95**  **(1.81-2.10)** | **1.72**  **(1.62-1.84)** | **1.26**  **(1.11-1.44)** | **1.15**  **(1.09-1.22)** | **4.29**  **(3.32-5.54)** | 1.01  (0.82-1.25) |
| Obesity | **0.88**  **(0.82-0.94)** | **1.09**  **(1.04-1.14)** | **1.14**  **(1.05-1.24)** | **1.16**  **(1.13-1.20)** | 1.00  (0.77-1.31) | 0.94  (0.81-1.08) |
| Mood disorder | **1.33**  **(1.26-1.40)** | **1.37**  **(1.32-1.43)** | **1.10**  **(1.02-1.18)** | **1.44**  **(1.40-1.48)** | **1.29**  **(1.03-1.61)** | 1.08  (0.95-1.22) |
| Any contraception | **1.14**  **(1.09-1.20)** | **1.05**  **(1.01-1.09)** | **1.20**  **(1.13-1.28)** | **1.07**  **(1.04-1.10)** | 1.05  (0.87-1.26) | **1.13**  **(1.02-1.25)** |
| Ectopic pregnancy^†^ | **0.80**  **(0.68-0.93)** | 1.01  (0.90-1.13) | **0.66**  **(0.54-0.83)** | 1.03  (0.94-1.13) | 0.68  (0.34-1.33) | 0.90  (0.64-1.26) |
| Non-ectopic pregnancy^†^ | **0.77**  **(0.74-0.82)** | **0.85**  **(0.82-0.89)** | **0.78**  **(0.73-0.83)** | **0.89**  **(0.86-0.91)** | 0.96  (0.78-1.18) | **0.85**  **(0.75-0.95)** |

^*^ Excluding procedures performed within 42 days of a birth; from multivariable Poisson models adjusted for age category, year of procedure, race/ethnicity, region, MediCal program, baseline months eligibility (log transformed), same-day ablation, health care utilization in the 2 years pre-procedure (i.e., claims related to abdominal pain or other gastrointestinal symptoms, genitourinary pain, menorrhagia, non-abdominal pain, pelvic inflammatory disease, pelvic pain, fibroids, mood disorder, obesity, pregnancy history, use of any contraceptive method, Charlson Comorbidity Index) presented if significant at p<0.05;

^†^ compared to no pregnancy during baseline period
